# Combined small angle X-ray solution scattering with atomic force microscopy for characterizing radiation damage on biological macromolecules

**DOI:** 10.1186/s12900-016-0068-2

**Published:** 2016-10-27

**Authors:** Luca Costa, Alexander Andriatis, Martha Brennich, Jean-Marie Teulon, Shu-wen W. Chen, Jean-Luc Pellequer, Adam Round

**Affiliations:** 1 ESRF, The European Synchrotron, 71 Avenue des Martyrs, Grenoble, 38000 France; 2 MIT, 77 Massachusetts Ave., Cambridge, 02139 MA USA; 3 Univ. Grenoble Alpes, 71 Avenue des Martyrs, Grenoble, 38044 France; 4 CNRS, IBS, 71 Avenue des Martyrs, Grenoble, 38044 France; 5 CEA, IBS, 71 Avenue des Martyrs, Grenoble, France; 6 European Molecular Biology Laboratory, 71 Avenue des Martyrs, Grenoble, 38000 France; 7 Unit for Virus Host-Cell Interactions, Univ. Grenoble Alpes-EMBL-CNRS, 71 Avenue des Martyrs, Grenoble, 38000 France; 8 Faculty of Natural Sciences, Keele University, Keele, Staffordshire UK; 9 Present Address: CBS, Centre de Biochimie Structurale, CNRS UMR 5048-INSERM UMR 1054, 29, Rue de Navacelles, Montpellier, 34090 France; 10 Present Address: European XFEL GmbH, Holzkoppel 4, Schenefeld, 22869 Germany

**Keywords:** *β*-Amylase, Tobacco mosaic virus, Small angle x-ray scattering (SAXS), Atomic force microscopy (AFM), Radiation damage

## Abstract

**Background:**

Synchrotron radiation facilities are pillars of modern structural biology. Small-Angle X-ray scattering performed at synchrotron sources is often used to characterize the shape of biological macromolecules. A major challenge with high-energy X-ray beam on such macromolecules is the perturbation of sample due to radiation damage.

**Results:**

By employing atomic force microscopy, another common technique to determine the shape of biological macromolecules when deposited on flat substrates, we present a protocol to evaluate and characterize consequences of radiation damage. It requires the acquisition of images of irradiated samples at the single molecule level in a timely manner while using minimal amounts of protein. The protocol has been tested on two different molecular systems: a large globular tetremeric enzyme (*β*-Amylase) and a rod-shape plant virus (tobacco mosaic virus). Radiation damage on the globular enzyme leads to an apparent increase in molecular sizes whereas the effect on the long virus is a breakage into smaller pieces resulting in a decrease of the average long-axis radius.

**Conclusions:**

These results show that radiation damage can appear in different forms and strongly support the need to check the effect of radiation damage at synchrotron sources using the presented protocol.

## Background

The most recent step forward in structural biology for characterizing large molecular assemblies is the integration of several complementary techniques to reach the goal of determining structures at atomic level. In this frame, it is essential to combine information from a variety of physical and chemical origins thus providing a solid basis to understanding molecular function. This recent development is known as integrative structural biology [1].

Among the different available techniques, small-angle X-ray scattering (SAXS) presents unique advantages. SAXS applied to dilute solutions of proteins is a long established technique in structural biology. It gives ensemble reciprocal space information on the size and shape of macromolecules [2–5]. While the reconstruction of 3D models of proteins from solution scattering data is common, it is an ill-posed problem and typically requires additional constraints such as the maximal distance between two points in a sample *D*_*max*_ [6–9]. SAXS data is sensitive to oligomerization or aggregation of biological samples. For example, radiation-induced aggregation has been observed with SAXS data for lysozyme, but without any change in folding topology [10]. Irreversible X-ray induced damage, essentially due to free radical formation in the sample at synchrotron sources, are a current limitation of SAXS experiments and often increase the amount of material needed [11] or require radiation protectant such as glycerol or cryo-cooling [12]. However, evaluation of post-SAXS experiment radiation damage on proteins is rarely performed because the allowable doses are highly sample-dependent, and must be determined on a case-by-case basis. A protocol to investigate such radiation damage at SAXS beamline is suggested in this work and makes use of the imaging capability of the atomic force microscope at nanometer resolution.

Atomic force microscopy (AFM) is beginning to make a large impact in the field of structural biology [13–17]. AFM uses a sharp tip located beneath a micro-cantilever that scans across sample molecules usually deposited on flat mineral substrates (e.g. Mica). It can give real space information about the size and shape of particles as well as their physical properties and behaviour in the measurement conditions. Typically, topographical resolution can reach sub-nanometer range when characterizing flat samples [15, 18–20] but it rises up to the nanometer range when measuring isolated biomolecules. The main advantage of AFM resides in its exceptional signal-to-noise ratio where the imaging of a single isolated particle is enough to determine its particular dimensions using standard or improved image processing methods [21]. Moreover, one of the main advantages of an AFM over scanning electron microscopy or transmission electron microscopy is that the sample can be kept in physiological conditions while imaging, such as a liquid buffer for proteins [22], a shared advantage with the SAXS technique. Complementarity between SAXS and AFM techniques allows cross validation thereby increasing the reliability and confidence in the results, and to obtain additional information such as electrochemical properties of a macromolecule based on its binding with surfaces for AFM images. However, to date there are only a few studies which combine these two techniques [23–27].

Here, we describe a protocol for the combined acquisition of bioSAXS and AFM data from the same sample with minimum delay taking advantage of the ESRF user facilities for both techniques. By using remaining (unexposed) sample from the bioSAXS acquisition and diluting it to the required concentration, depositing it onto an atomically flat surface for AFM imaging, the AFM data collection is achieved with no additional sample required over that needed for bioSAXS. Indeed, as little as 1 *μ*L of the sample solution left in the sample changer was diluted 1000 times to a concentration suitable for AFM. With this method, the amount of solution leftover from SAXS is sufficient for many AFM images, which can be very useful in cases where each *μ*L of solution takes large amounts of time and resources to produce. It is shown that AFM is a useful tool to evaluate the effects of radiation damage by evaluating changes to physical characteristics and electrochemical behaviour of irradiated samples. Such effects result in an increase of the apparent size or in a decrease of the average particle radius due to breakage. It is also shown that the AFM output can be employed as a constraint to interpret SAXS data, reducing ambiguity in the SAXS output. To evaluate the performance of the AFM-SAXS combination, two standard systems have been used: *β*-Amylase from sweet potato and the tobacco mosaic virus (TMV), a long rod shape plant virus.

## Methods

### Sample preparation

*β*-Amylase is a tetrameric enzyme of ≈ 200 kDa which catalyzes the hydrolysis of maltose units (two glucose units) for starch. The known crystal structure of *β*-Amylase (PDB code 1FA2) [28] was used for comparison with experimental data. According to the reconstituted tetrameric structure of *β*-Amylase, the computed maximum bounding box of C *α* atoms has a size of 12.4 × 12.4 × 7.5 nm ^3^ and a radius of gyration of 4.14 nm (all atoms). A 5.5 mg/mL solution of *β*-Amylase protein from sweet potato (Product Number A8781, Sigma-Aldrich, St. Louis, MO, USA) was prepared by adding 3.75 mL of Tris equilibration buffer (50 mM TRIS-HCl, 100 mM KCl, pH 7.5, 5 *%* v/v glycerol, Sigma-Aldrich) and filtering with a 0.2 *μ*m filter. The final concentration was verified using a NanoDrop spectrophotometer at OD _280_ (*ε*_1 *%*_=17.7) [29]. For AFM, the solution was further diluted in Tris equilibration buffer without glycerol.

TMV forms a hollow rod-like structure of about 300 nm length and 19 nm diameter. The crystal structure of TMV was determined and refined by X-ray fiber diffraction [30]. The organization of TMV assembly has been widely studied using imaging techniques such as electron microscopy, AFM, and X-ray microdiffraction [31–34], and it is a common model system for image processing [35]. The regularity of the TMV structure simplifies the comparison of results out of single-image analysis on AFM images with those of other techniques. TMV particles were prepared as previously described [36]. The concentration was determined by spectrophotometric measurement at OD _260_ (*ε*_0.1 *%*_=3.1) at a value of 26 mg/mL. TMV dilution for AFM and SAXS was performed in deionized water.

### Small-angle x-ray scattering (SAXS)

SAXS data collection was performed on BM29 at the ESRF [37]. 50 *μ*L of sample solution was loaded into the automatic sample changer, with 40 *μ*L used for the actual experiment. Scattering data of samples and buffers was acquired at one frame per second for ten seconds while sample was flowing through the capillary using the flow mode of the automated sample changer [38]. For AFM imaging of radiation damaged samples, the exposed samples were recuperated after exposure and followed the same protocol as the non exposed samples to facilitate comparison. The total flow time was 18 seconds. The X-ray beam energy was 12.5 keV and the beam size was 700 × 700 *μ*m. The detector distance was 2.864 m. Data was collected at 20 °C. Intensity was normalized to absolute units using background-corrected water. The available *q*-range ($q= \frac {4\pi }{\lambda } \sin \theta $) was 0.025 nm ^−1^ to 4.8 nm ^−1^. Data reduction was done using the standard tools at BM29 yielding the 1D subtracted curves and initial processing to give feedback to the experiment [39, 40]. Extrapolation to zero concentration and determination of the model independent parameters (*R*_*g*_ (radius of gyration), *I*_0_ (Intensity at *q* = 0), molecular mass, etc.) and cross-sectional Guinier analyis done using PRIMUS [41, 42]. Comparison of known structures to the experimental data (*β*-Amylase) was done using OLIGOMER [41]. Fitting of geometric models (TMV) was done with Genfit [43]. Computation of model intensities was done using CRYSOL [44] while p (*r*) (pair distribution) analysis and cross-section pair-distribution using GNOM [42].

Radiation damage investigations were performed testing several irradiation times: 1) one second exposure, 2) standard exposure which corresponds to a total exposure of 10 s while flowing, 3) 5 min exposure, 4) 30 min over-exposure. For both latter exposures, the sample was flowing continuously back and forth through the beam. We have collected a total of 10 patterns for standard exposure and 6 patterns for 5 min and 30 min exposures. The comparison between SAXS and AFM data has been limited to standard, 5 min and 30 min exposures.

### Atomic force microscopy (AFM)

AFM images were recorded in amplitude modulation (tapping) mode in liquid [45] with photothermal excitation on Cypher and piezo-dither excitation mode on a MFP3D (both Asylum Research, Santa Barbara, USA). Cantilevers used are the MSNL (triangular lever F, k = 0.6 N/m, *F*_*q*_=30 kHz in liquid, Bruker) and Olympus AC40 (k ≈ 0.1 N/m, *F*_*q*_=25 kHz in liquid, Olympus). Scan sizes were 10 *μ*m ×10 *μ*m, 5 *μ*m × 5 *μ*m, or 1 *μ*m × 1 *μ*m.

The scan rate was 2 lines per second, the image size was either 512 lines and 512 points per line or 256 lines and 256 points. The atomically flat surfaces were cleaved mica, cleaved mica ion-exchanged with Nickel, and cleaved highly ordered pyrolytic graphite (HOPG). Pre-treated mica was prepared by placing 40 *μ*L of 10 mM NiCl _2_ on a cleaved mica disk, incubating for 10 minutes, then rinsing with water and drying with nitrogen [46].

To deposit *β*-Amylase protein onto a surface, 40 *μ*L of 1/1000 diluted solution (4 *μ*g/mL) were placed on the surface and incubated for 15 min. The surface was then rinsed with 1 mL of buffer and covered with 40 *μ*L of buffer for imaging in liquid.

To deposit tobacco mosaic virus (TMV), 40 *μ*L of 1/1000 diluted TMV solution (26 *μ*g/mL) was placed on the surface and incubated for a full hour before rinsing. The surface was then rinsed with 1 mL of water and covered with 40 *μ*L of water for imaging in liquid.

To obtain a representative collection of AFM images, three different 10 *μ* *m* ^2^ areas were imaged randomly on a given sample. Within each area, three 5 *μ* *m* ^2^ random areas were chosen from which to characterize proteins. If necessary, 1 *μ* *m* ^2^ scans were made for areas of particular interest to provide higher resolution images. Image data treatment, such as flattening, was performed using Gwyddion [47] and/or DeStripe [48].

### Statistical evaluation of isolated particles in AFM images

Previously flattened AFM height images were used in Gwyddion. When necessary additional flattening was performed to further reduce stripe noise. A grain particle analysis was performed with Gwyddion using the automated thresholding method of Otsu for all images except images of *β*-Amylase when deposited on bare mica in which case a classical thresholding method of 11–32 *%* was used instead. Grain size distribution was recorded using the major semiaxis of equivalent ellipse (called here long-axis radius). Ellipse was chosen instead of circle due to the elongated shape of TMV. Consequently, the same numerical measure has been used for *β*-Amylase and TMV. However, control data did not reach exactly a long-axis radius of 150 nm for TMV (as should be expected from its structure). This is likely due to the fact that the automated ellipsoid fit performed in Gwyddion does not distinguish TMV particles that are touching with each other. Average values were obtained from at least two AFM images for control and over-exposed conditions using the top 2 peaks of the grain particle distribution. Observations made with this criteria would also be valid if we took the top peak of the distribution. The reason to combine the top 2 peaks is that in several distributions, there is not a single major top peak and thus the “correct” value corresponding to such a distribution ought to be the average of nearby peaks. Values reported on figures are average +/− standard deviations.

### Protocol for combining SAXS with AFM

When planning combined experiments, it is important to consider the effects of X-ray exposure, both short and long term, related to the bioSAXS experiment [11, 12]. BioSAXS data is routinely checked for short term variations during exposure and shows there is no variation on the length scales (low resolution size and shape) and time scales (1–10 s at dedicated facilities). Remeasured samples (hours/days after initial exposure) can show significant differences. It is for this reason and the favorable highly dilute state (1000 × dilution from bioSAXS) required for AFM that samples were measured by SAXS and only the remaining sample volume (not aspirated by the sample changer) was used for AFM. For studying the effect of radiation damage, samples exposed to the X-ray beam for measurements were recuperated and analyzed by AFM alongside the non-exposed controls. Due to the time needed to prepare the sample for AFM, the time between the two experiments was 20 minutes for *β*-Amylase and an hour for TMV.

## Results

### *β*-Amylase

**Fig. 1:**
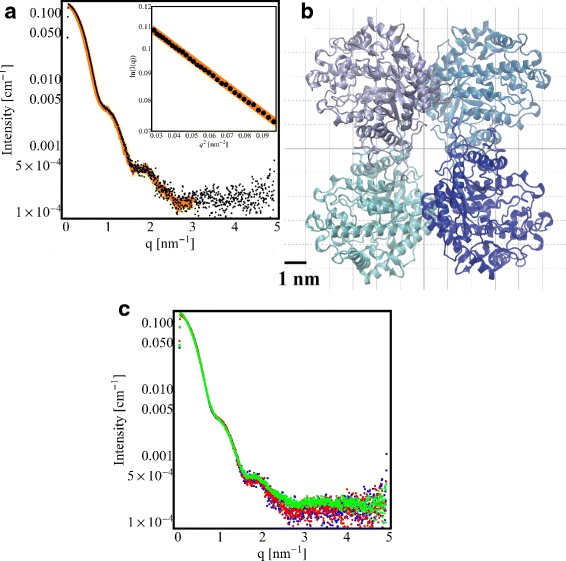
*β*-Amylase SAXS data. **a** SAXS curve of *β*-Amylase (*black dots*) obtained after a standard exposure with fit to a mixture of monomers and tetramers (*orange line*). Inlay: Guinier plot and associated linear fit at low- *q* ^2^ (*q* *R*_*g*_ < 1.3). **b** Model of tetrameric *β*-Amylase (pdb entry 1FA2) [28]. The bounding *box size* of tetrameric *β*-Amylase is 12.4 × 12.4 × 7.5 nm ^3^. **c** SAXS data for different exposures time: standard, 5 min and 30 min exposures are plotted in *blue*, *red* and *green*, respectively; very small variations with the exposure time can be observed

The background corrected SAXS curve of *β*-Amylase is shown in Fig. 1 a. The mean radius of gyration of the solution is 4.2 nm. To verify that the *β*-Amylase solution consisted primarily of tetrameric *β*-Amylase complexes, the scattering of the *β*-Amylase in solution was compared with the theoretical scattering of monomeric, dimeric, and tetrameric *β*-Amylase proteins using the program OLIGOMER [41]. Using atomistic models of the protein monomer, dimer, and tetramer, the program fits the theoretical scattering of all three to the observed solution scattering and determines their ratio. *β*-Amylase was determined to consist of about 94 *%* tetramers and 6 *%* monomers (Fig. 1 a) without any contribution from dimeric forms (*χ* ^2^=4.19).

AFM images were obtained with *β*-Amylase adsorbed on hydrophilic mica in liquid environment using the amplitude modulation (tapping) mode. No significant adsorption of *β*-Amylase has been observed on hydrophobic graphite (Data not shown). When *β*-Amylase was deposited onto mica, a uniform distribution of particles was observed, with an average height of 2 nm and an average long-axis radius of 1.87 nm which is smaller than expected from the diameter of *β*-Amylase crystal structure (Fig. 1 b). According to molecular sizes observed with AFM, it is likely that only monomers of *β*-Amylase are imaged by AFM.

**Fig. 2:**
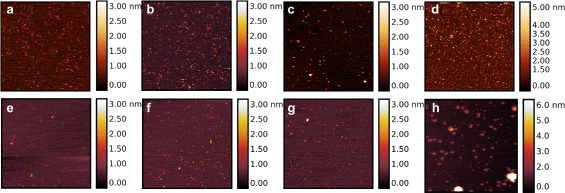
AFM imaging of *β*-Amylase using tapping mode in liquid environment. *Top row* (**a**, **b**, **c**, **d**) corresponds to images obtained when *β*-Amylase was deposited on Nickel pre-treated mica whereas the *bottom row* (**e**, **f**, **g**, **h**) corresponds to bare mica. Non-irradiated *β*-Amylase is shown in (**a**, **e**) whereas increasing exposure to X-ray beam is shown in (**b**, **f**) for 10 s exposure, (**c**, **g**) 5 min exposure, (**d**, **h**) 30 min exposure. The scan size is 1 *μ*m with each line made of 512 pixels. A clear increase in height (and diameter) can be easily seen upon increase exposure (radiation damage) with no apparent differences between control and standard exposure (10 s) time. A total of 13 AFM images have been used for statistical analysis representing a total of 3693 and 948 particles measured on nickel pre-treated mica and bare mica, respectively

Because the surface of mica is negatively charged, two experimental conditions were used to image irradiated *β*-Amylase : bare mica or mica pre-treated with NiCl _2_ solution. Except for native *β*-Amylase, samples were collected after SAXS beamline exposure. Figure 1 c reports SAXS data at different exposure times for *β*-Amylase. Results of AFM imaging are found in Fig. 2.

**Fig. 3:**
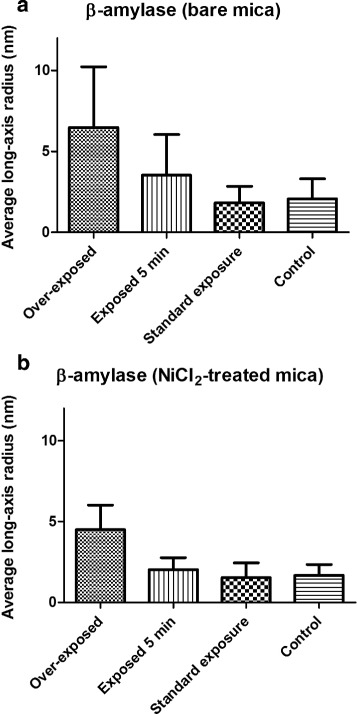
Average *long-axis* radius of *β*-Amylase estimated from their distribution in AFM images. Particles were identified using the threshold or the Otsu’s method when *β*-Amylase was deposited on bare mica (**a**) or Nickel pre-treated mica (**b**). Control represents *β*-Amylase that was not exposed to X-ray. Standard exposure is about 10 s whereas over-exposed corresponds to a 30 min exposure to X-ray. *Long-axis* radii were determined with standard parameters of the Grain distribution section of Gwyddion. Upon increase exposure time in X-ray beam, a slight increase in the long-axis radius of *β*-Amylase is observed which could be interpreted as aggregation of *β*-Amylase monomers or consolidation of *β*-Amylase tetramer after radiation damage (see text). The number of identified particles on bare mica was 139, 122, 457 and 230 for over-exposed, exposed 5 min, standard exposure and control, respectively; whereas on nickel pre-treated mica the number of particles was 1877, 136, 1453 and 277 for over-exposed, exposed 5 min, standard exposure and control, respectively

Figure 3 shows that increasing X-ray beam exposure provokes an enlargement of the long-axis radius of isolated particles (see Methods for definition of long-axis radius). It can be easily seen that standard exposure time for *β*-Amylase does not modify significantly the shape of *β*-Amylase as imaged by AFM. However, a continuous increase of the long-axis radius can be observed when *β*-Amylase was exposed during 5 min to the X-ray beam. Over-exposure of *β*-Amylase in the X-ray beam provokes a dramatic increase in the long-axis radius of AFM imaged *β*-Amylase. The increase in the mean size upon X-ray beam exposure is systematically observed in all experiments both on bare and Nickel pre-treated mica.

**Fig. 4:**
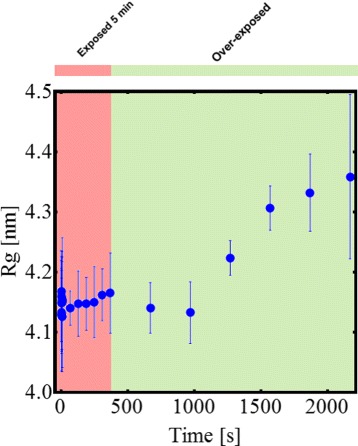
Evolution of the radius of gyration for the *β*-Amylase upon X-ray exposure obtained from SAXS data. While there is no significant increase for low exposure time, *R*_*g*_ increases once proteins are over-exposed: this is consistent with the AFM results presented in Fig. 3

Table 1 reports all structural parameters evaluated at different exposures time. Interpretation of SAXS data on various X-ray beam exposure time of *β*-Amylase indicates that there is no significant change in size, especially the radius of gyration, of irradiated *β*-Amylase (Fig. 4 obtained treating data presented in Fig. 1 c). This is in contrast to AFM measurements (Figs. 2 and 3) for which the magnitude of change of the long-axis radius of *β*-Amylase due to X-ray beam exposure is large. In AFM, the *β*-Amylase long-axis radius upon irradiation is close to the expected size of the *β*-Amylase tetramer according to its X-ray structure.

**Table 1:** SAXS data-collection and scattering-derived parameters

Data-collection parameters				
Instrument	ESRF BM29			
Beam Geometry	0.7 mm ×0.7 mm at sample			
Wavelength (nm)	0.099			
*q* range (nm ^−1^)	0.05-4.95			
Exposure time	1 s per frame			
Concentration, *β*-amylase	5.5 mg/mL			
Concentration, Tobacco Mosaic Virus	26 mg/mL			
Temperature (K)	293			
Structural Parameters, *β*-amylase				
Exposure time	1 s	10 s (standard)	5 min	30 min
*I*_0_ (cm ^−1^) [from Guinier]	0.135 ± 0.001	0.135 ± 0.001	0.133 ± 0.1	0.142 ± 0.001
*R*_*g*_ (nm) [from Guinier]	4.14 ± 0.06	4.14 ± 0.06	4.16 ± 0.06	4.35 ± 0.13
*I*_0_ (cm ^−1^) [from *P*(*r*)]	0.134	0.135	0.135	0.132
*R*_*g*_ (nm) [from *P*(*r*)]	4.09	4.10	4.12	4.22
*D*_*max*_ (nm)	1.10	1.13	1.20	1.30
Porod volume estimate (nm ^3^)	267	267	266	283
Structural Parameters, Tobacco Mosaic Virus				
Exposure time	1 s	10 s (standard)	5 min	30 min
*I*_0_ (cm ^−1^ x nm ^−1^)	0.312 ± 0.003	0.325 ± 0.003	0.330 ± 0.003	0.317 ± 0.003
[from cross-sectional Guinier]				
cross-sectional *R*_*g*_ (nm)	6.34	6.40	6.51	6.49
[from cross-sectional Guinier]				
*I*_0_ (cm ^−1^ x nm ^−1^)	0.278	0.284	0.289	0.279
[from cross-sectional *P*(*r*)]				
cross-sectional *R*_*g*_ (nm) [from cross-sectional *P*(*r*)]	5.89	5.89	5.88	5.87
cross-sectional *D*_*max*_ (nm)	18.0	18.0	18.0	18.0
Molecular-mass determination, *β*-amylase				
Exposure time	1 s	10 s (standard)	5 min	30 min
Partial specific volume (cm ^3^ *g* ^−1^)	0.724			
Contrast (*Δ* *ρ*×10^10^ *cm* ^−2^)	3.047			
Molecular mass *M*_*r*_ (kDa) [from I _0_]	168 ± 1	170 ± 1	167 ± 1	178 ± 1
Molecular mass *M*_*r*_ (kDa)	157	157	156	166
[from Porod Volume] [64]				
Calculated molecular weight according to sequence (kDa)	224			
Software employed				
Primary data reduction	BM29 online data analysis [65],			
	pyFAI [66], Primus [64]			
1D data processing	PRIMUS			
p(r) analysis	GNOM [42]			
Form factor fitting	GENFIT [43]			
Computation of model intensities	CRYSOL [44]			
Computation of volume fractions of mixtures	OLIGOMER [64]			

### TMV

**Fig. 5:**
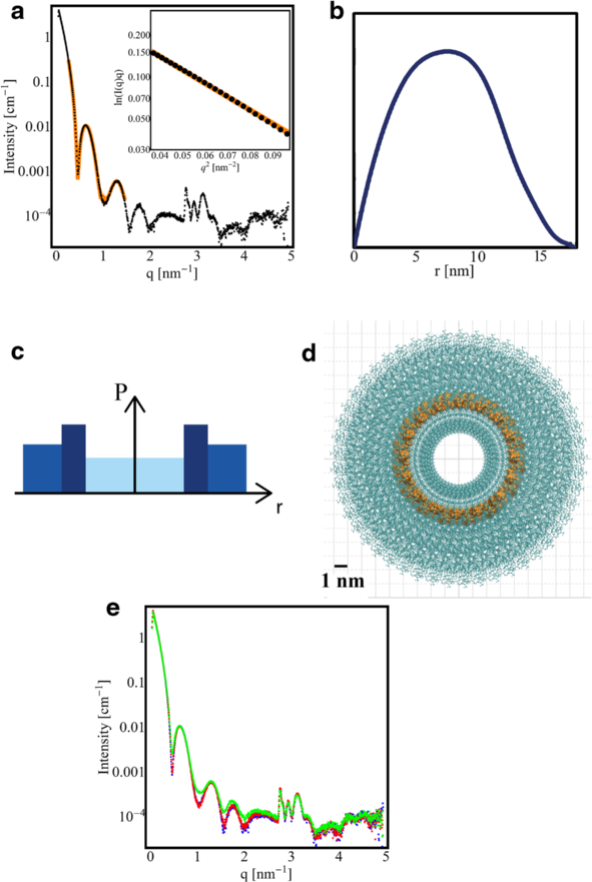
TMV SAXS data. **a** SAXS curve of TMV (*black dots*) obtained during a standard exposure with fit to a three shell cylinder model (*orange line*). Inlay: Cross-sectional Guinier plot and fit. **b** Radial pair distance distribution function of TMV using the virus diameter found by AFM as constrained for D _*max*_. **c** Schematic of the three-shell electron density distribution used as a model in **a**). **d** Atomistic model of the TMV cross-section with the RNA in *orange*. Based on pdb entry 1VTM. **e** SAXS data for different exposures time: standard, 5 min and 30 min exposures are plotted in *blue*, *red* and *green*, respectively

Crystal structure of TMV, as well as electron micrographs, indicate that TMV is about 300 nm long with a diameter of about 19 nm [35]. The background corrected SAXS cross sectional Guinier plot of TMV is shown in the inset of Fig. 5 a. The cross-sectional radius of gyration is found to be 6.34 nm. The low *q*-region of the curve can be fitted with a three shell cylinder model, using parameters comparable to those reported in the literature (Fig. 5 c) [49]. The peaks in the region of 3 nm ^−1^ can be attributed to fibre diffraction from the helical repeat of TMV (2.9 nm) [50, 51]. The cross-sectional pair distance distribution function shown in Fig. 5 b was calculated using *D*_*max*_=18 nm based on the virus height determined by AFM. It is rather symmetric, with its maximum at 7.5 nm. It is noteworthy that the AFM output has been used here as constraint for SAXS data treatment as it is conventionally done using NMR or electron microscopy data. Figure 5 e reports SAXS data at different exposure times. TMV SAXS patterns present very small variations in the *q*-range of 0.05 to 2 nm ^−1^. However, the structural parameters reported in Table 1 for TMV do not show any consistent variation with the exposure time.

**Fig. 6:**
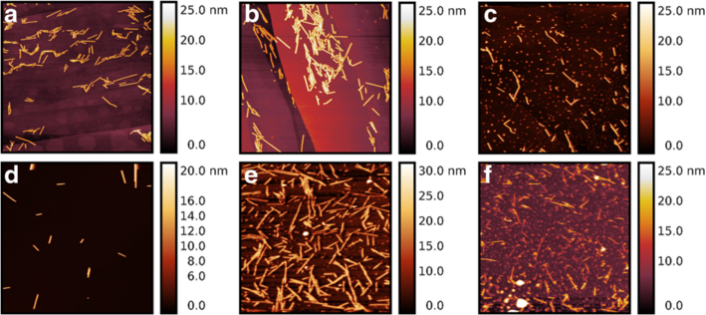
AFM imaging of TMV using tapping mode in liquid environment. *Top row* (**a**, **b**, **c**) is TMV deposited on HOPG whereas *bottom row* (**d**, **e**, **f**) corresponds to TMV deposited on Nickel pre-treated mica. Non-irradiated TMV is shown in (**a**, **d**). Increasing exposure to X-ray beam is shown in (**b**, **e**) for 10 s exposure, and (**c**, **f**) 30 min exposure. The scan size is 5 *μ*m with each line made of 512 pixels. A clear fragmentation of the 300 nm-long TMV can be observed upon increase in exposure time (radiation damage). To the contrary of *β*-Amylase (Fig. 2), even at standard exposure time, a beginning of fragmentation is observed for TMV. A total of 14 AFM images has been used for statistical analysis representing 6539 particles on HOPG at 10 *μ*m scan size, 2007 particles on HOPG at 5 *μ*m scan size, and 808 particles on nickel pre-treated mica

TMV particles were imaged with AFM after SAXS measurements both on freshly cleaved HOPG or pre-treated mica (Fig. 6 a–f respectively). On freshly cleaved mica no TMV could be detected (data not shown) whereas on Nickel-coated mica, TMV particles of about 17 nm height were detected (Fig. 6 d, e, f). The coverage was estimated at 16.9 *%* using a mask selecting all points higher than 5 nm. When deposited on HOPG (hydrophobic surface), TMV particles of similar height (17 – 18 nm) were observed with a surface coverage of 11.2 *%* (Fig. 6 a, b, c). The observed heights of TMV are similar to values reported in the literature when imaging TMV on mica [52].

**Fig. 7:**
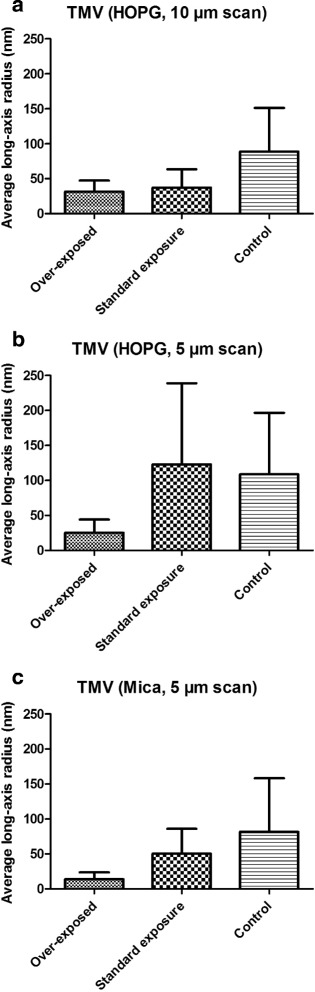
Average long-axis radius of TMV estimated from their distribution in AFM images. Particles were identified using the Otsu’s method when TMV was deposited on HOPG (**a**, **b**) or mica (**c**). AFM data have been acquired on unexposed samples (control), after a standard exposure as well as after 30 min exposure (over-exposed). Upon increasing exposure time in X-ray beam, a consistent decrease in the long-axis radius of TMV is observed which corresponds to a fragmentation of TMV particles upon radiation damage. The number of identified particles on HOPG 10 *μ*m was 3912, 2273 and 354 for over-exposed, standard exposure and control, respectively; on HOPG 5 *μ*m the number of identified particles was 1906, 68 and 33 for over-exposed, standard exposure and control, respectively; on mica the number of identified particles was 347, 157 and 304 for over-exposed, standard exposure and control, respectively

Effect of radiation damage is clearly seen in AFM images between control data (Fig. 6 a, d) and over-exposed data (Fig. 6 c, f) as well as standard exposure (Fig. 6 d, e) when TMV was deposited on the hydrophobic HOPG surface. Quantification of such effect is obtained by estimating long-axis radius measurements of TMV particles upon different X-ray beam exposure times (Fig. 7). By combining all AFM data, long-axis radius of TMV in over-exposed data is about 22 nm whereas the value for control data is about 93 nm. It can be seen that, to the contrary of *β*-Amylase, increasing exposure time on TMV lead to a reduction in its long-axis radius from 4 to 5 fold. A gradual and consistent decrease in TMV long-axis radius can be seen from control, to standard, and over-exposure. It can also be observed that the long-axis radius of control TMV is not 150 nm (perfect fit) but about 2/3 of this value. It is likely that the 2D image fitting algorithm performs poorly when TMV particle appears in bunch rather than well isolated (Fig. 6). The clear difference observed in AFM images of TMV on mica and HOPG suggests a clear physico-chemical change occurring for TMV upon radiation damage. Whereas native TMV tends to aggregate on HOPG, a more uniform adsorption on HOPG is observed upon X-ray beam exposure concomitantly with a reduction in TMV particle average length.

At the ESRF beamline, it is not possible to perform ultra-small angle X-ray scattering experiments. Consequently, changes in length of TMV could not be obtained using regular SAXS data due to large size of TMV (≈300 nm long).

## Discussion

SAXS scattering provides reliable characterization of the average structural properties of biological macromolecules by measuring the scattering curve and interpreting it to determine model-independent structural parameters of molecules. Although 3D reconstructions of shape of macromolecules from scattering curves are possible, they are often not unique. Moreover, SAXS as a technique is able to visualize a wide range of dimensions depending on the X-ray energy and the angular range observed, but the maximum size observable for any given experiment is limited. For the standard setup at BM29 a *q*-range of 0.025 to 5 nm ^−1^ can be observed which corresponds to a longest particle dimension of approximately 250 nm. As the long axis of tobacco mosaic virus (TMV) is in the order of 300 nm, this dimension could not be measured directly in the standard configuration of BM29. Fortunately, the observed scattering of the TMV is dominated by the circular cross-section of the cylinder, featuring the coat protein and RNA strand allowing direct comparison between SAXS and AFM. The AFM experiments confirmed the rod-like structure of the TMV sample with a cross-section of 17–18 nm. The cross-sectional radius of gyration was calculated as 6.3 nm assuming a rod-like structure. For a homogeneous disc of radius $R, R = \sqrt {2} R_{g}= 8.9$ nm, which is in direct agreement with the diameter of 18 nm reported in literature [53]. It should be emphasized that the calculation of (cross-sectional) pair distance distribution function requires to find the correct maximum distance *D*_*max*_. In this work, this value was obtained from real space measurements with the AFM allowing a direct access to this parameter, thereby greatly reducing the ambiguity of the analysis. In the case of TMV, using the 18 nm diameter determined by AFM indeed allowed us to calculate the cross-sectional pair distance distribution function without bias.

Radiation damage [54] have been mostly investigated in X-ray crystallography where it was observed that radiation damage on proteins starts with the reduction of metal centers followed by elongation/scission of disulfide bonds and then decarboxylation of Asp and Glu side chains [55]. Moreover, such decarboxylation of acidic amino acids is also observed due to radiation damage with electron microscopy [56].

AFM imaging of single molecules has already been used to observe protective effect of ascorbic acid against double-strand breaks in DNA generated by reactive oxygen species [57]. In addition, AFM has also been coupled with Dynamic Light Scattering technique to help in understanding consequences of radiation-induced conformational change in chromatin structure. It was found that even at low dose (< 0.5 Gy) chromatin shows radiation damage as evidence by a change in hydrodynamic size that was likely due to single-strand breaks in DNA [58]. In SAXS, radiation damage most often present itself as aggregation [59]. Even with sample flow enable, radiation damage in lysozyme, evidenced by an increase in radius of gyration (*R*_*g*_), still occurs as early as 250 ms exposure time [11]. At increasing dose on lysozyme, an increase in *R*_*g*_ has been observed in relation to radiation damage [60]. Combining SAXS with UV/Visible absorption spectra revealed change in protein solution due to X-ray radiation on bovine serum albumine (BSA) as shown by an increase of *R*_*g*_ from 3.3 to 5 nm [61]. However, it was also found that early effect of radiation damage was an increase of molecular size without any significant unfolding suggesting that radiation damage observed on BSA was compatible with the presence of radical activities [61]. Reduction in radiation damage has been obtained using Cryo-SAXS [12] or using time-resolved SAXS [62]. Besides, fast detection readout allows collection of SAXS before radiation damage occur [60].

In our study, AFM imaging of isolated molecules of *β*-Amylase revealed a tripling in size upon over-exposure to X-ray beam. From this result, two hypotheses are possible: agglomeration of several *β*-Amylase monomers or a tightening of *β*-Amylase tetramer upon X-ray exposure so that when imaged on mica the tetrameric form of *β*-Amylase is now stable and better preserved than *β*-Amylase without X-ray exposure. The second hypothesis appears more likely due to SAXS observation that no significant change in *R*_*g*_ was observed upon over X-ray exposition of *β*-Amylase (Fig. 4 and Table 1), and that *β*-Amylase remains mostly tetrameric. Besides, it has been shown that one consequence of irradiation damage in synchrotron SAXS experiment was a change of the protein surface due to radical attacks leading to a greater attraction between lysozyme molecules and causing aggregation: a mechanism that could also be envisaged to multimeric proteins such as *β*-Amylase [10]. The current resolution of AFM does not allow imaging at the atomic scale on isolated proteins to identify more precisely what is the mechanism of such increase in size. In particular, knowing the convolution effect due to tip broadening in AFM images, it is not possible to attribute *β*-Amylase native tetramers on AFM controlled images (long-axis radius of about 3 nm). The only possible explanation is that only smaller structures are observed, mostly monomers whose presence is also detected by SAXS, for non irradiated *β*-Amylase while, upon X-ray exposure, AFM images show an expected size of tetrameric *β*-Amylase. At the moment, it is not possible to speculate about the presence of crosslink in *β*-Amylase upon X-ray exposure, as the resolution of AFM imaging does not allow such level of details.

Finally, it is striking that AFM imaging can indirectly distinguish between two conformations of tetrameric *β*-Amylase: native and X-ray over-exposed while SAXS data does not make a significant distinction.

Radiation damage in TMV particle is different from *β*-Amylase essentially due to the high aspect/ratio of TMV which is a rod-like of 300 nm long. TMV, as most plant viruses, are very stable molecular constructs that can resist harsh storage condition (dessicated) for several years. TMV is consequently a perfect sample for studying radiation damage as no degradation is expected to occur when deposited on mica [36]. Upon increasing exposure time in X-ray beam, a breaking of TMV is consistently seen. In this case, radiation damage on TMV resemble closely that obtained on DNA, i.e. breaking into smaller parts.

It is noteworthy that the imaging substrate surface has a significant importance in AFM. This is for instance brought forward by the comparison between bare mica and nickel pre-treated mica in this study (AFM data not shown). Indeed, TMV adsorption is more efficient on nickel pre-treated mica than on simple mica. However, no substrate is ideal due to the apparent contradictory requirement of strong fixation of biomolecules on a surface with low deformation of adsorbed molecules.

Surface charges of mica may affect the shape of deposited single molecules. For instance, a height reduction of 2 nm is observed when TMV is deposited on mica and imaged in air, while the height difference of TMV when imaged in liquid is close to 0.7 nm (manuscript submitted). However, such reduction in height has never been observed to be concomitant with a reduction of TMV length as observed in this study when TMV is exposed to X-ray beam.

Another difference between TMV and *β*-Amylase, is that with TMV radiation damage are detected with short exposure time whereas in *β*-Amylase radiation damage are mostly visible upon over-exposure time. However, a common behavior between *β*-Amylase and TMV upon X-ray beam exposure is their apparent change in molecular surface properties. Although it is only suggested for *β*-Amylase, it is clearly observed for TMV. Indeed, when native TMV is deposited on hydrophobic surface (HOPG) there is non uniform binding of TMV on HOPG whereas, upon X-ray beam exposure, TMV displays an increased uniformity in adsorption with HOPG. Because breakage of long TMV particles into smaller pieces, damaged TMV now exposes hidden buried surfaces. From the TMV X-ray structure, such hidden surfaces are known to be rather hydrohobic explaining the sudden increased affinity of irradiated TMV on HOPG. A clear benefit of AFM imaging is observed, first by looking at individual molecules, and second at global properties when changing imaging substrates. Consequently, if reasonable protein binding is observed on HOPG, it could be recommended to use hydrophobic surfaces for imaging X-ray exposed molecules, and thus detect easily the presence of radiation damage by looking at variation of protein binding on HOPG. Such apparent change in molecular surface properties has been already observed in case of lipid model membranes deposited onto silicon substrates in an *in-situ* X-ray - AFM combined experiment [63]. Both X-ray Reflectometry and AFM showed a deacrease of the membranes surface coverage after exposure to X-ray.

## Conclusion

The combination of SAXS and AFM can be applied to a variety of different macromolecules and sample surfaces depending on characterization needs and sample properties. Taking advantage of the flexible user access to both the dedicated bioSAXS beamline (BM29) and ESRF surface science laboratory, these experiments can be undertaken on the same visit to the ESRF. AFM imaging requires around two hours and owing to the high dilution factor from SAXS to AFM no additional sample is needed for AFM in addition to SAXS. While SAXS provides rapid characterization of the average properties of a sample, AFM can be used to verify the homogeneity of the sample and provide measurements at the single particle level. As AFM gives direct measurement of single particles it is possible to use the AFM results as additional constraints for modeling purpose thereby extending the possibilities to interpret the SAXS data and reduce ambiguity in the results. The use of combined SAXS-AFM in one experimental visit is facilitated by the presented protocol which enables cross validation, and increased confidence in the conclusions which can be drawn from the experiments. Furthermore, combination of SAXS-AFM is well adapted to study effect of radiation damage on various type of biological samples. Radiation damage is a very complex process and can produce either a change of the protein surface or a breakage of long biological particles, as it has been shown in this work for *β*-Amylase and TMV, respectively.

## Abbreviations

AFM: Atomic force microscopy
EM: Electron microscopy
ESRF: European synchrotron radiation facility
HOPG: Highly ordered pyrolitic graphite
NMR: Nuclear magnetic resonance
SAXS: Small angle x-ray scattering
TMV: Tobacco mosaic virus
